# The activin-follistatin anti-inflammatory cycle is deregulated in synovial fibroblasts

**DOI:** 10.1186/s13075-019-1926-7

**Published:** 2019-06-10

**Authors:** Magnus Diller, Klaus Frommer, Berno Dankbar, Ingo Tarner, Marie-Lisa Hülser, Lali Tsiklauri, Rebecca Hasseli, Michael Sauerbier, Thomas Pap, Stefan Rehart, Ulf Müller-Ladner, Elena Neumann

**Affiliations:** 1 Department of Rheumatology and Clinical Immunology, Justus Liebig University Giessen, Campus Kerckhoff, Bad Nauheim, Benekestr: 2-8, 61231 Bad Nauheim, Germany; 2 Institute of Experimental Musculoskeletal Medicine, University Hospital Münster, Münster, Germany; 3 Department of Plastic, Hand and Reconstructive Surgery, BGU Frankfurt, Frankfurt, Germany; 4 Department of Orthopaedics and Trauma Surgery, Agaplesion Markus Hospital, Frankfurt, Germany

**Keywords:** Activin A, Follistatin, Synovial fibroblasts, Rheumatoid arthritis

## Abstract

**Background:**

Activin A and follistatin exhibit immunomodulatory functions, thus affecting autoinflammatory processes as found in rheumatoid arthritis (RA). The impact of both proteins on the behavior of synovial fibroblasts (SF) in RA as well as in osteoarthritis (OA) is unknown.

**Methods:**

Immunohistochemical analyses of synovial tissue for expression of activin A and follistatin were performed. The influence of RASF overexpressing activin A on cartilage invasion in a SCID mouse model was examined. RASF and OASF were stimulated with either IL-1β or TNFα in combination with or solely with activin A, activin AB, or follistatin. Protein secretion was measured by ELISA and mRNA expression by RT-PCR. Smad signaling was confirmed by western blot.

**Results:**

In human RA synovial tissue, the number of activin A-positive cells as well as its extracellular presence was higher than in the OA synovium. Single cells within the tissue expressed follistatin in RA and OA synovial tissue. In the SCID mouse model, activin A overexpression reduced RASF invasion. In human RASF, activin A was induced by IL-1β and TNFα. Activin A slightly increased IL-6 release by unstimulated RASF, but decreased protein and mRNA levels of follistatin.

**Conclusion:**

The observed decrease of cartilage invasion by RASF overexpressing activin A in the SCID mouse model appears to be mediated by an interaction between activin/follistatin and other local cells indirectly affecting RASF because activin A displayed certain pro-inflammatory effects on RASF. Activin A even inhibits production and release of follistatin in RASF and therefore prevents itself from being blocked by its inhibitory binding protein follistatin in the local inflammatory joint environment.

**Electronic supplementary material:**

The online version of this article (10.1186/s13075-019-1926-7) contains supplementary material, which is available to authorized users.

## Background

Activin A is a disulphide-linked homodimer composed of two β_A_-subunits (β_A_β_A_) of inhibin A, which itself is formed by an α- and β_A_-subunit. The heterodimer consisting of an α- and β_B_-subunit is called inhibin B. Accordingly, two other forms of activin can be distinguished: activin B (β_B_β_B_) and activin AB (β_A_β_B_) [1, 2]. Both activins and inhibins are members of the transforming growth factor β (TGF-β) superfamily. Their role in the hypothalamic-pituitary-gonadal axis is well known [3], but activins are also linked to inflammatory and fibrotic processes [4]. In marrow stromal cells, activin A is upregulated by tumor necrosis factor α (TNFα), lipopolysaccharide (LPS), interleukin-1α (IL-1α), and IL-1β [5, 6]. In vivo experiments with animals confirmed the systemic activin A release in circulation after LPS injection [7, 8]. The activin A increase was biphasic and followed by the release of the activin A-binding protein follistatin [7, 9]. The effect of activin A has been described as pro- as well as anti-inflammatory, depending on the examined cell type or cellular activation state. For example, the release of pro-inflammatory cytokines of activated macrophages could be blocked by activin A [10], but quiescent macrophages were stimulated by activin A to produce pro-inflammatory cytokines [11, 12].

The role of activin A and follistatin in chronic autoinflammatory disorders is not fully understood. There is evidence that activin A promotes allergic diseases [13] and inflammatory bowel disease [14], whereas these effects could be blocked by follistatin. In inflamed joints affected by rheumatoid arthritis (RA), activin A was expressed by synovial fibroblasts (RASF) in the synovial membrane and upregulated in the RA synovium compared with osteoarthritis (OA) or normal joint tissues [15, 16]. Elevated activin A levels were also found in the synovial fluid of RA compared to OA patients [15]. RASF proliferation was elevated by activin A and reduced by follistatin [16]. Pro-inflammatory cytokines like TGF-β, TNFα, and IL-1β increased the activin A production in RASF [16]. In a carrageenan-induced mouse arthritis model, follistatin injection reduced macrophage infiltration into the synovium and inhibited proteoglycan erosion [17].

Due to the clues pointing towards a role of activin A in RA, the aim of the study was to investigate the role of activin A and follistatin in the inflammatory and matrix degrading response of RASF and the known feedback loop between activin A and follistatin described for other cell types.

## Methods

### Tissues and cells

RA/OA synovium and OA cartilage were obtained during knee replacement surgeries (Agaplesion Markus Hospital). RA patients fulfilled classification criteria of the American College of Rheumatology [18, 19]. Human OA cartilage with macroscopically intact surface was cut [20] and in part snap-frozen for hematoxylin/eosin staining (H/E), for which areas with normal histological structure were used. Sample collection of the synovium and cartilage was approved by the local ethics committee (Justus Liebig University Giessen), and all patients gave written informed consent.

Synovium samples were snap-frozen, used for paraffin-embedding, or digested (1 h Dispase-II-solution, 0.1 ml/ml, PAN-Biotech, Germany) [21] for fibroblast isolation. Cells were cultured up to passage 5 in DMEM (PAA-Laboratories, Germany) containing 10% heat-inactivated fetal calf serum (FCS, Sigma-Aldrich, Germany), 1 U/ml penicillin/streptomycin, and 1 mM HEPES (PAA-Laboratories) at 37 °C and 10% CO_2_ [21].

### Immunohistochemistry

Formalin-fixed 5 μm paraffin sections were deparaffinized, and antigen retrieval was performed with 4 M hydrochloric acid (follistatin, β2-microglobulin) or proteinase K (vimentin). The tissue was permeabilized with Triton X-100 and endogenous peroxidases blocked with 0.3% H_2_O_2_ in 100% methanol. After blocking with 10% dry milk, the slides were incubated overnight with primary antibodies in 2.5% BSA at 4 °C: goat anti-human/mouse/rat polyclonal activin A (AF338, R&D, Germany), mouse anti-human monoclonal follistatin (MAB669, R&D, Germany), goat anti-human polyclonal ACVR2A (A8081) and ACVR1B (A2455) (both Sigma-Aldrich, Germany), mouse anti-human monoclonal β2-microglobulin (ab54810, Abcam, UK), and mouse anti-human monoclonal vimentin (M7020, Dako, USA). Slides were incubated 30 min with secondary antibodies (Histofine, Medac), and color development was performed with AEC substrate (Vector Laboratories, USA). For snap-frozen tissues, 5 μm acetone-fixed sections were used with the same procedure.

### Activin A overexpression

Recombination vector pAdLox (digested with EcoR1) was used to generate adenoviral vectors with either the full-length activin A (for: 5′-CTGTCTTCTCTGGACAACTC-3′, rev: 5′-GCAGGGCCTTTTAAAAAGGC-3′) or the GFP sequence inserted as a control. The adenoviral vectors were provided as a courtesy of the University of Pittsburgh. Based on previous experiments, a multiplicity of infection (MOI) of 100 was used for RASF or OASF transduction [21]. The absence of virus in the supernatants after one passage was confirmed by real-time PCR.

### SCID mouse model

Female, 6-week-old Crl-scidBR mice (Charles River, Germany) were kept under pathogen-free conditions with water and food ad libitum. Animal experiments were performed in accordance with the German Animal Welfare Act and approved by the local government authorities, RP Oberfranken, Germany, 621-2531.1-13/03. Animals underwent surgery with implantation of 1.5 × 10^5^ SF together with healthy areas of human OA cartilage in a carrier matrix (Gelfoam, Pfizer, USA) with up to four cartilage implants per animal [20]. SCID mice were sacrificed after 60 days, and implants removed, snap-frozen, stained (H/E), and used for scoring [20, 22, 23].

### Synovial fibroblast stimulation

RASF or OASF were cultured for 48 h. The medium was replaced and cells stimulated with IL-1β or TNFα (10 ng/ml each; R&D) with or without activin A/AB (15 ng/ml; R&D) or follistatin (500 ng/ml; R&D) for 15 h. Supernatants were centrifuged and stored at − 20 °C. As control, stimulation was performed under serum-free conditions.

### Protein measurements

Cytokines, matrix-degrading proteinases (MMP), and growth factors were measured by enzyme-linked immunosorbent assay (ELISA, R&D) or Luminex analysis for IL-6, IL-1β, TNFα, IL-10, VEGF, IL-12p40, GM-CSF, IFNγ, IL-8, IL-4, IL-2, and IL-5 using the Bead-based multiplex kit (R&D).

### RNA extraction and cDNA synthesis

RASF were harvested and total RNA extracted (RNeasy Mini Kit, Qiagen, Germany). Remaining DNA was removed using the RNase-free DNase Set (Qiagen). RNA concentrations were quantified (Ribogreen RNA quantification kit, Molecular Probes, Netherlands, or Nanodrop system, Thermo Fisher) and RNA stored at − 80 °C.

cDNA was synthesized using 150 ng RNA, 5 mM Tris-HCl (pH 8.3, 25 °C), 50 mM KCl, 1 mM MgCl_2_, 0.5 mM spermidine, 1 mM dithiothreitol, 1 mM each dNTP (Roche, Germany), A260 unit random primer (Roche), 1.6 U/μl RNase inhibitor (Roche), and 1.3 U/μl AMV reverse transcriptase (Promega, Germany). Conditions were 25 °C 10 min, 42 °C 60 min, and 99 °C 5 min. cDNA was stored at − 20 °C.

### Polymerase chain reaction (PCR)

Real-time PCR was performed (LightCycler system, Roche) using SYBR Green detection including melting curve analysis. 18S rRNA served as an endogenous control. Primer efficiencies were tested by the standard curve method (*E* = 10^–1/slope^, *E* = 2.00 ± 0.05 was considered acceptable). PCR mixture includes 2 μl cDNA or water, 0.5 μM each primer, 10 μl 2xQuantiTect® SYBR® Green PCR Master Mix (Qiagen), and MgCl_2_ according to primer efficiency. PCR products were subjected to a melting curve analysis. Data were analyzed using the LightCycler analysis software (Roche). Primers include follistatin for: 5′-GTCGGGATGTTTTCTGTCCAG-3′ and rev: 5′-TGGCATAAGTGGCATTGTCAC-3′ (4 mM MgCl_2_, *T*_ann_ = 50 °C).

For evaluation of activin A receptor type 1 (*ACVR1*) and activin A receptor type 2A (*ACVR2A*), standard PCR was performed using the Titan One-Tube RT-PCR system (*T*_ann_ = 55 °C, Roche) followed by agarose gel electrophoresis (1%). Primers include *ACVR1* for: 5′-AGCATCAACGATGGCTTCCA-3′, rev 5′-AGTGCTGTCTCCAACATTGG-3′; *ACVR2A* for: 5′-GGTGTACAGGCATCACAAGA-3′, rev: 5′-CCAAGAGACCACATTAGCCT-3′; 18S for: 5′-TCAAGAACGAAAGTCGGAG-3′, rev: 5′-GGACATCTAAGGGCATCACA-3′).

### Western blot

RASF (*n* = 3) were pre-incubated with serum-free medium for 2 h before stimulation with activin A for 10 min and lysed after stimulation (10 mM Tris, 150 mM NaCl, 1 mM EDTA, 0.2% sodium deoxycholate, 1% NP-40 and protease/phosphatase inhibitors (Roche)). Western blotting was performed with antibodies against total Smad2 (#5339, CST, UK) and phosphorylated Smad2 (#3101, CST). For detection, secondary goat anti-rabbit HRP-conjugated antibodies (Dako) and the ECL system (GE Healthcare, USA) were used. Antibodies against activin A (mouse anti-human, R&D) and ACVR2A (A8081, goat anti-human polyclonal, Sigma) were detected using secondary anti-mouse HRP-conjugated antibodies (goat anti-mouse; donkey anti-goat, Santa Cruz). Cyclophilin B served as the loading control.

### Statistics

All data are presented as arithmetic mean ± standard deviation (SD). For comparisons with a single control group, one-way ANOVA followed by Dunnett’s post hoc test was performed. Multiple comparisons among several groups were performed by one-way ANOVA followed by Bonferroni’s post hoc test. For comparison of two groups with different treatments and increasing stimulation duration, two-way ANOVA followed by Bonferroni’s post hoc test was performed. The assessment of significance level for pair-wise comparisons was calculated by a Student two-tailed *t* test and Mann-Whitney *U* test. *p* values < 0.05 were considered significant. Statistical calculations were performed and graphics created using GraphPad Prism.

## Results

### Detection of follistatin and activin A and their receptors on synovial fibroblasts

**Fig. 1:**
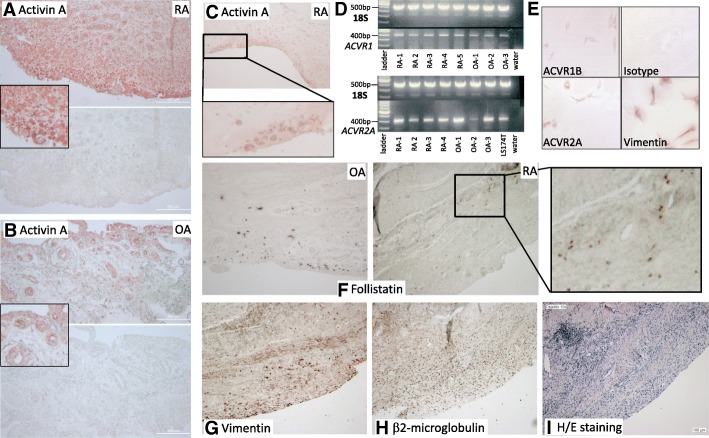
Detection of follistatin and activin and their receptors on synovial fibroblasts. **a** Activin A staining of RA synovial tissue compared to **b** OA synovial tissue (representative stainings, *n* = 4 each). **c** Activin A staining at the site of cartilage invasion in RA (*n* = 4). **d** mRNA of *ACVR1* was detectable in all 5 RASF and 3 OASF and mRNA of *ACVR2A* in all 4 RA- and 3 OASF; here, LS174T cells are shown as a positive control. Negative control: water instead of RNA. 18S rRNA served as the loading control. **e** Immunocytochemistry for ACVR2A and ACVR1B protein confirmed the presence of both receptors on cultured RASF. Positive control: mesenchymal marker vimentin, negative control: matched isotype control. **f** Follistatin expression was limited to single cells in RA and OA synovial tissue (*n* = 3) when compared to the **g** vimentin staining showing the distribution of mesenchymal cells including synovial fibroblasts as well as **h** β2-microglobulin, an MHC class I subunit expressed by almost all nucleated cells. **i** H/E staining of the tissue. 100-fold magnification

In hyperplastic RA synovium, the number of activin A expressing cells and presence of the secreted protein in the extracellular matrix surrounding the cells was higher compared to OA (*n* = 4) (Fig. 1a, b). Cells at sites of cartilage invasion also expressed activin A (Fig. 1c). Activin A receptor expression (*ACVR1* and *ACVR2A*) was comparable on cultured RA- and OASF by PCR (Fig. 1d) and by immunocytochemistry for ACVR2A and 1B in RASF (Fig. 1e). In addition, the effect of activin A on the activin A receptor type 2A (ACVR2A) expression was evaluated by western blot showing that stimulation with activin A did not significantly alter ACVR2A expression in RASF (Additional file 1). Synovial tissue evaluation by immunohistochemistry showed that only few cells expressed follistatin (RA and OA, *n* = 3 each Fig. 1f) compared to the total number of cells and vimentin-positive fibroblasts (Fig. 1g, h). The synovial lining layer was mostly negative for follistatin (Fig. 1f).

### Effect of activin A on RASF-mediated cartilage invasion in vivo

**Fig. 2:**
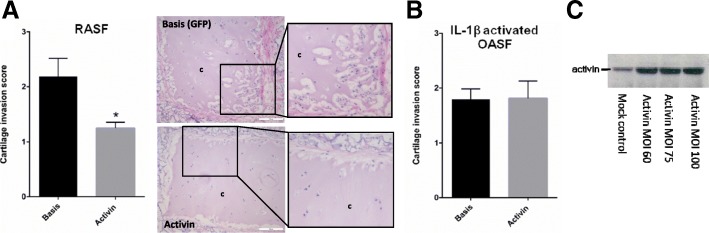
Effect of activin A on cartilage invasion. **a** RASF were coimplanted together with healthy human cartilage into SCID mice. After 60 days, the invasion of RASF into cartilage was reduced by adenoviral activin A overexpression in comparison to control (*n* = 4 animals with *n* = 14 implants per group). By way of example, implants with GFP-transduced RASF (Basis) and for activin A overexpressing RASF are shown (c = cartilage). **b** OASF were activated with IL-1β to induce cartilage invasion, and activin A overexpression did not change IL-1β-induced OASF cartilage invasion (*n* = 5 implants without activin, *n* = 10 implants with activin). **c** Overexpression of activin A in RASF was confirmed by western blot. An MOI of 100 was used for implantation with mock-treated cells showing baseline activin A protein expression in RASF

In the SCID mouse model, RASF invaded coimplanted human cartilage as published previously [22]. However, activin A overexpression in RASF reduced RASF invasion into cartilage compared to GFP controls (*p* < 0.05) (Fig. 2a). Due to the limited capacity of OASF to invade cartilage [22, 24], OASF were pre-activated with IL-1β and then coimplanted. Activin A overexpression in OASF did not affect IL-1β-induced invasion. Activin A overexpression was confirmed by western blot prior to implantation (Fig. 2c).

### Activin A suppresses follistatin

**Fig. 3:**
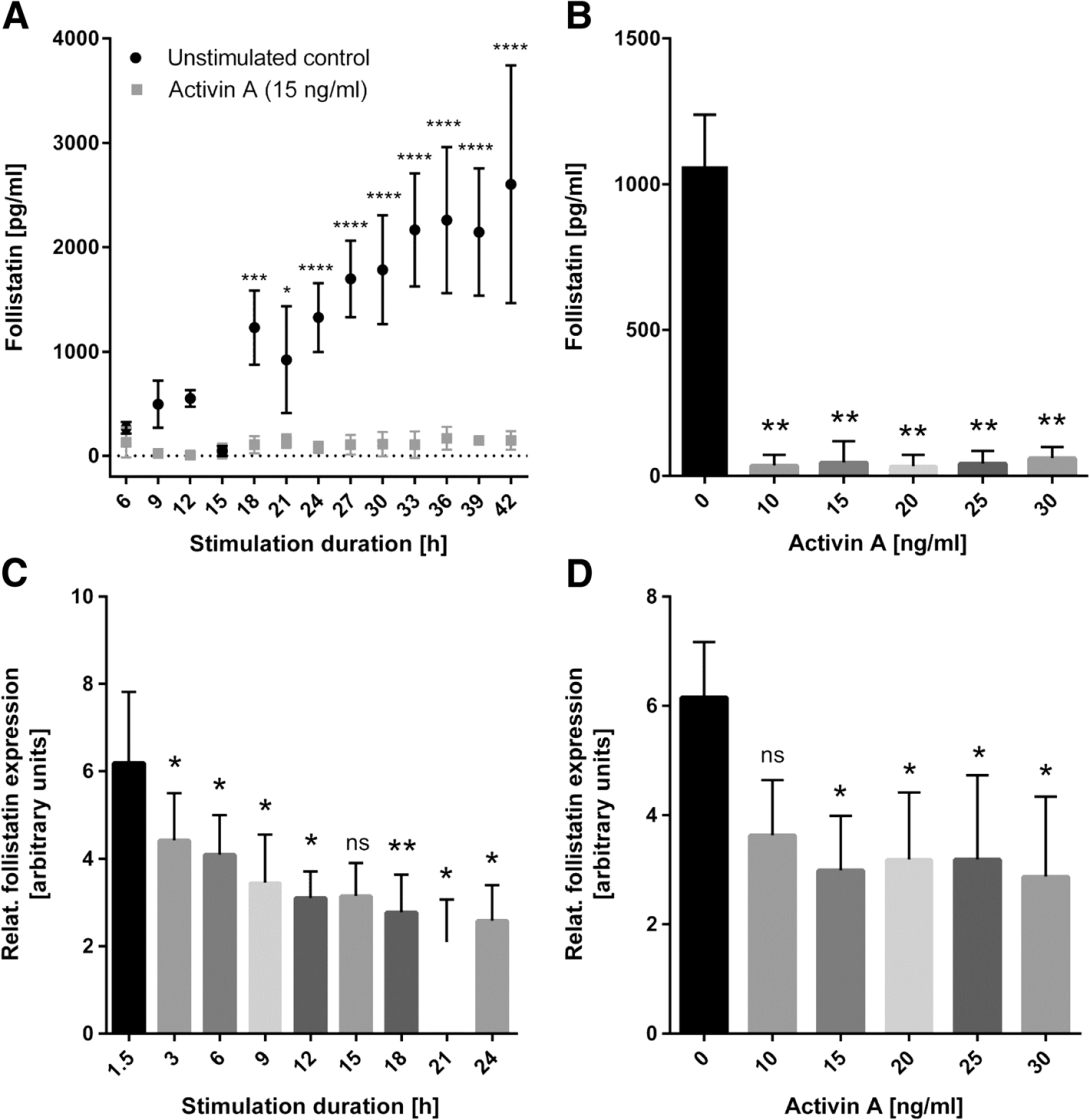
Synovial fibroblast stimulation with activin A suppresses follistatin. **a** Activin A suppressed follistatin protein release in RASF at all time points from 6 to 42 h (15 ng/ml activin A, *n* = 4). **b** Concentrations of 10–30 ng/ml activin A had the same suppressive effect on follistatin protein (shown for 15 h, *n* = 4). **c** Stimulation of RASF with activin A (15 ng/ml, *n* = 5) from 1.5 to 24 h resulted in a reduced expression of follistatin RNA. **d** When stimulating RASF for 3 h with 0 to 30 ng/ml activin A, a significant reduction of follistatin RNA expression was observed (15 h, *n* = 4). **c**, **d** 18S rRNA measurement served as the normalization control

Kinetics of RASF (*n* = 5) stimulated with activin A at 15 ng/ml showed suppression of follistatin protein secretion over time (6–42 h stimulation, Fig. 3a). At all time points, the use of activin A concentrations of 10–30 ng/ml suppressed follistatin protein expression (shown for 15 h, Fig. 3b). The activin A-mediated follistatin suppression decreased at concentrations below 5 ng/ml activin A (Additional file 2). The reduction of follistatin RNA expression was confirmed by real-time PCR for up to 24 h (Fig. 3c) and after using different activin A concentrations (Fig. 3d). Due to the kinetics, 15 ng/ml activin A and 15 h for stimulation were selected for further experiments.

### Alteration of inflammatory parameters by activin A

**Fig. 4:**
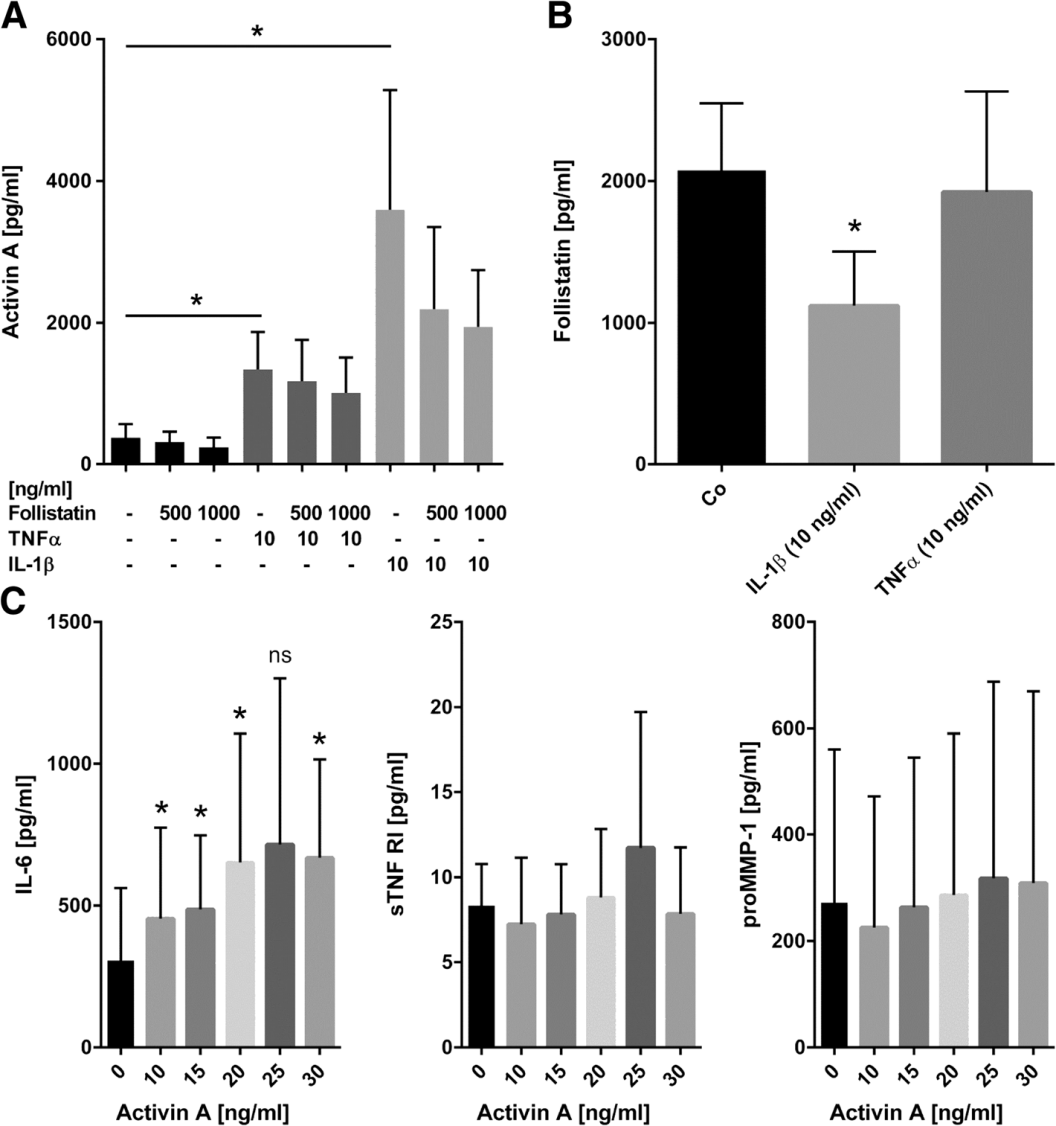
Effect of inflammatory factors on activin A and follistatin in RASF. **a** Activin A protein release was induced by TNFα and to a higher extent IL-1β. The IL-1β-induced activin A level could be reduced by follistatin but not TNFα-induced activin (*n* = 7 RASF). **b** Follistatin level was reduced by IL-1β but not other pro-inflammatory stimuli (*n* = 5 RASF). **c** IL-6 showed highly individual baseline levels in different patients. IL-6 (*n* = 6), proMMP-1 (*n* = 7), or sTNF-RI (*n* = 4) levels were not induced by more than 2-fold by activin A

Activin A was induced by IL-1β and to a lower extent by TNFα in RASF (*n* = 7, Fig. 4a, *p* < 0.05). Follistatin production in RASF was not altered by stimulation with 10 ng/ml TNFα, whereas 10 ng/ml IL-1β decreased the follistatin release from 2075 ± 474 to 1121 ± 380 pg/ml (0.54-fold, *p* < 0.05, Fig. 4b). Other factors such as RANKL, OPG, or oncostatin M (an IL-6 signaling pathway inducer) did not alter follistatin levels (data not shown). Activin A between 10 and 30 ng/ml slightly induced IL-6 (maximum 2.2-fold with 30 ng/ml activin A, *p* < 0.05, Fig. 4c). Other factors such as proMMP-1 and soluble TNF receptor I (sTNF-RI, Fig. 4c), MMP-13, MMP-3, TGF-β, IL-1 receptor antagonist (IL-1ra), GM-CSF, and IFNγ were detectable but not altered by activin A or follistatin (data not shown). IL-10, IL-4, IL-1β, IL12p40, and TNFα were close to or below the detection limit of the ELISA and not induced by activin A or follistatin (data not shown).

### Effect of activin A on synovial fibroblasts under inflammatory conditions

**Fig. 5:**
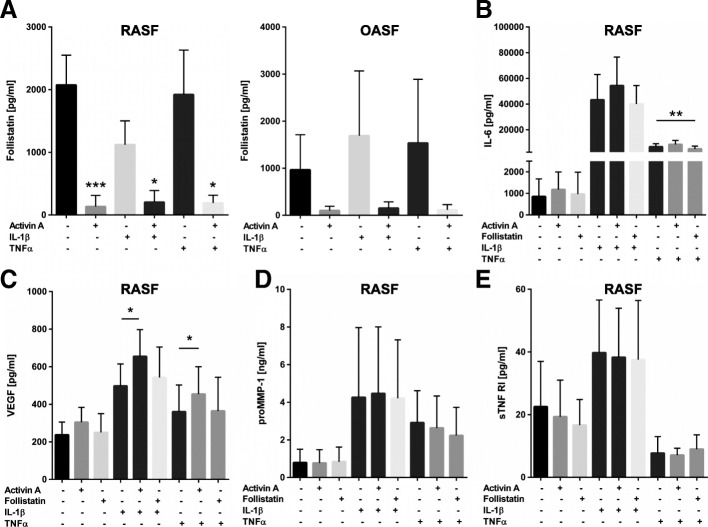
Effect of activin A on synovial fibroblasts under inflammatory conditions. **a** Activin A suppressed follistatin in RA- and OASF independently of the presence of pro-inflammatory stimuli. **b** IL-6 was induced by TNFα and to a stronger extent by IL-1β (10 ng/ml each, *n* = 6) but follistatin or activin A did not induce more than 2-fold changes with activin A, and similar results were observed for VEGF (**c**, *n* = 6) as well as for proMMP-1 but without an effect on proMMP-1 when adding activin A (**d**, *n* = 7). sTNF-RI was induced by IL-1β but reduced by TNFα without an effect of activin A/follistatin (**e**, *n* = 4)

IL-1β or TNFα were added to RASF in combination with activin A. In all settings, addition of activin A completely suppressed follistatin in RASF as well as reduced follistatin in OASF without reaching statistical significance. The decrease was independent of the presence of the pro-inflammatory stimuli (Fig. 5a). Factors such as IL-6, proMMP-1, and VEGF were not affected by activin A or follistatin (Fig. 5b–d). However, activin A increased the VEGF release of RASF stimulated with IL-1β or TNFα (*p* < 0.05, Fig. 5c). Activin A or follistatin had no effect on sTNF-RI release (Fig. 5e). Other parameters such as MMP-13, TGF-β, IL-1Rα, GM-CSF, and IFNγ were detectable, but only effects of IL-1β and/or TNFα but not of activin A or follistatin were visible (data not shown). IL-10, IL-4, IL-1β, IL12p40, and TNFα were close to or below the detection limit of the ELISA. Results from stimulations under serum-free conditions were comparable (not shown).

### Stimulation with follistatin or activin AB

**Fig. 6:**
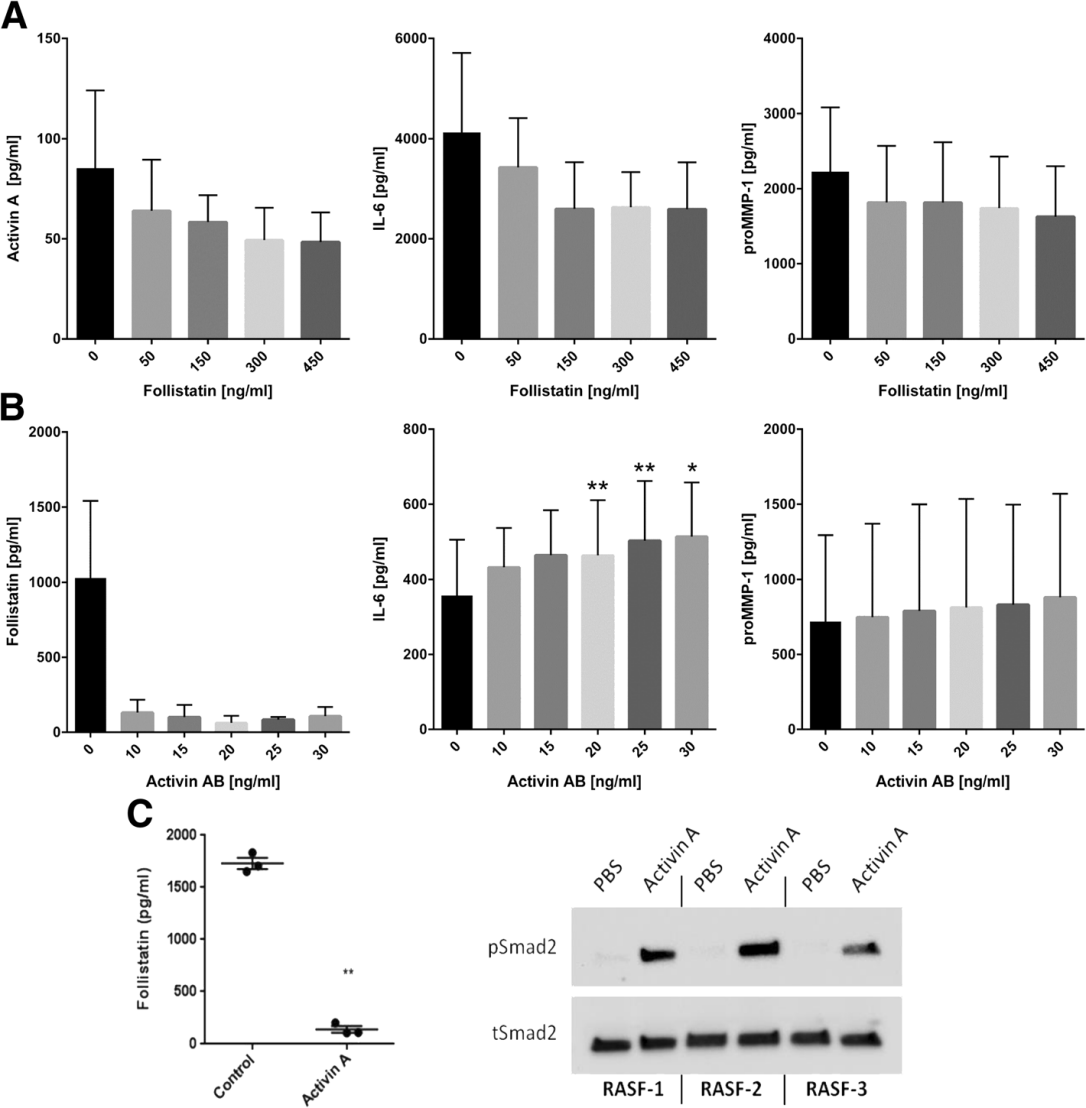
Effect of follistatin and activin AB on RASF and activin-induced Smad signaling. **a** Stimulation of RASF with follistatin showed a less than 2-fold reduction of activin A, IL-6, and proMMP1 (*n* = 3). **b** Stimulation with activin AB showed a significant follistatin reduction whereas IL-6 was less than 2-fold induced and no effect on proMMP-1 could be observed (*n* = 3). **c** In RASF (*n* = 3), activin A-mediated suppression of follistatin was confirmed in parallel to Smad2 phosphorylation. In these RASF, activin A strongly induced phosphorylation of Smad2. Results of two-tailed paired *t* test and mean ± SE are shown

When stimulating RASF with follistatin, activin A concentrations were slightly reduced. However, reduction was less than 2-fold and did not reach significance. Similarly, IL-6 and proMMP-1 levels were not attenuated significantly (Fig. 6a). Stimulation with activin AB led to similar results compared to activin A with a strong reduction of follistatin and low or no induction of other parameters including IL-6 and proMMP-1 (Fig. 6b).

### Confirmation of activin A-induced signaling

Phosphorylation of Smad2, a well-known signaling pathway of the TGF-β superfamily, could be detected by western blot (*n* = 3, *p* < 0.01, Fig. 6c). In parallel to the Smad2 phosphorylation, actvin A-mediated suppression of follistatin was confirmed (Fig. 6c).

## Discussion

As previously described, the activin A levels in synovial fluid and its expression in the synovium are elevated in RA [15, 16]. Indeed, activin A expression in the RA synovium is higher compared to OA, indicating a possible role in RA pathogenesis and in inflammatory processes and/or neoangiogenesis. The activin A concentrations measured in vivo in inflamed joints (up to 39 ng/ml) have shown to block in vitro the IL-6-induced proliferation of 7TD1 B lymphoid cells, the phagocytic activity of monocytic M1 cells, and the fibrinogen production in HepG2 [25]. These findings indicate an anti-inflammatory action of activin A in the context of RA. Indeed, in the SCID mouse model, the invasive behavior of RASF overexpressing activin A was decreased compared to GFP controls, whereas the activin A overexpression in IL-1β activated OASF did not influence the invasion score. Our findings support the anti-inflammatory action of activin A in RA in vitro, but on the other hand, studies focusing on RASF have shown that activin A increased RASF proliferation [16]. Accordingly, the antagonist follistatin inhibited RASF proliferation induced by IL-1β [16]. Based on the decreased invasive behavior of RASF in the SCID mouse model, we examined the effect of activin A and follistatin on cytokine and MMP levels of RASF.

We could show that activin A and AB increased the IL-6 release of RASF and contributed to the observed accelerated proliferation of RASF stimulated by activin A in vitro [16]. Nevertheless, the pathophysiological relevance in vivo of the induced release of IL-6 by activin A by approximately 2-fold has to be questioned due to the high amounts of IL-6 present within inflamed joints. Moreover, IL-1β increased the IL-6 release about 1000-fold. The levels of other inflammatory factors and MMPs were not affected showing that activin A does not act on RASF as a potent pro- or anti-inflammatory cytokine at least for the parameters evaluated in this study. Consequently, we could not observe a relevant reduction of cytokines or MMPs in activated or non-activated RASF by application of follistatin. However, activin A increased the VEGF release of RASF treated with IL-1β or TNFα, and therefore, activin A may contribute to neoangiogenesis and capillary permeability, effects known to be mediated by VEGF [26].

The major source of activin A is synovial fibroblasts and CD68+ mononuclear cells [16]. Our data are in line with previous findings showing an increased activin A release by stimulation with IL-1β and TNFα [16]. These findings confirm the role of RASF as a source of activin A in inflamed joints. Activin A was also shown to promote RANKL-induced osteoclast formation, and therefore, activin A produced by RASF could indirectly contribute to bone erosion [27–29]. The decrease of RASF invasion in our SCID mouse model cannot be explained by the observed effects in vitro*.* However, they could be explained as a result of an altered expression of activin A and follistatin in RASF affecting local cells in the more complex system in vivo.

Follistatin has mainly been described as an anti-inflammatory component inhibiting experimental induced allergic asthma and inflammatory bowel disease in mice by blocking activin A [14, 30]. In acute inflammatory reactions, the source of the follistatin release following the increase of activin A remains unclear [7, 9]. Possible cells producing follistatin in a negative feedback loop as an answer to activin A are liver cells as shown for the human hepatocellular carcinoma cell line HepG2 [31]. Interestingly, we showed that follistatin expression was limited to single cells in RA synovium. Indeed, in vitro, activin A decreased follistatin production and release by RASF independently of the duration up to 3 days. This effect was also independent of the activin A concentration, and even low doses were able to block the follistatin release as well as reduce mRNA levels. This behavior does not seem to be specific for RA synovial fibroblasts because OASF also showed the reduced follistatin release suggesting a fibroblast-specific effect. Even though the effects of follistatin or activin A on RASF regarding, e.g., IL-6 seems to be negligible, in the local inflammatory joint environment, the decrease of follistatin levels could possibly play a role in RA and OA through the missing inhibition of activin A effects on immune cells such as activated tissue macrophages. Therefore, activin A prevents itself from being blocked by inhibition of the release and gene expression of follistatin. The suppression of follistatin induced by activin A could also explain the limited follistatin expression in RA synovium. The effect is probably mediated by Smad signaling as shown for RASF in our study and since Smad signaling is a well-known pathway activated by the TGF-β superfamily [32].

Our data indicate a decrease of follistatin release after stimulation of RASF with IL-1β but not TNFα. The observed 0.54-fold reduction of follistatin by 10 ng/ml IL-1β may be due to the increased production of activin A induced by IL-1β itself. TNFα increased the release of activin A but to a lesser extent compared to IL-1β, which could explain the difference.

**Fig. 7:**
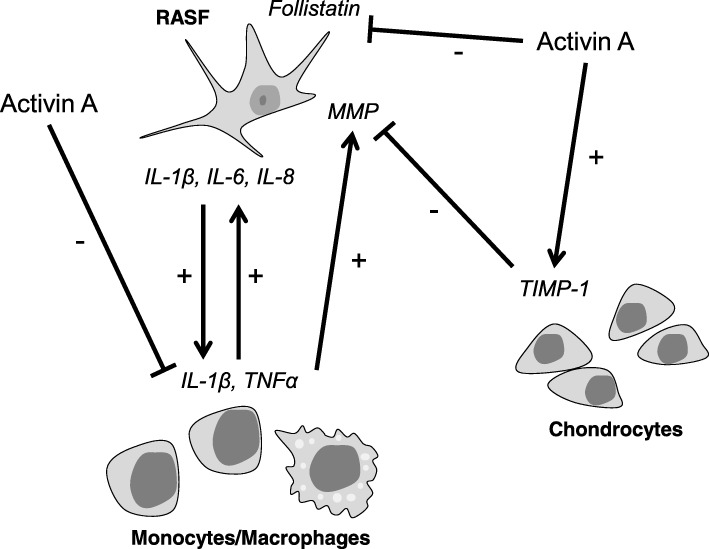
Possible mechanisms involved in reduced invasiveness of activin A overexpressing RASF in the SCID mouse model. RASF and monocytes/macrophages activate each other specifically at sites of cartilage invasion by secretion of pro-inflammatory cytokines (e.g., IL-1β, TNFα produced by macrophages, IL-6 produced by fibroblasts). IL-1β increases the invasiveness of RASF in the SCID mouse model. Activin A decreases the IL-1β production in monocytes/macrophages. In contrast, activin A increases TIMP-1 expression in chondrocytes. Additionally, the expression of follistatin, an antagonist of activin A, is reduced in RASF by activin A

Taken together, there is a discrepancy between the observed effect of activin A on RASF in vitro and the reduced invasion of RASF overexpressing activin A in SCID mice. SCID mice are characterized by an impaired immune system with severe lymphopenia but unaltered monocytes and macrophages [33]. Therefore, in the SCID mouse model, the interactions of monocytes/macrophages, RASF, and chondrocytes within the cartilage are key players in the invasion process of RASF. Interestingly, activin A was described to induce the production of TIMP-1 (tissue inhibitor for metalloproteinases-1) in human chondrocytes [34] and decreased the production of IL-1β in activated U-937 cells and in mouse macrophages activated with LPS [10, 35]. Pap et al. showed that IL-1β contributes to the invasion of RASF [36]. Consequently, the decreased invasion by RASF overexpressing activin A could possibly be mediated by the reduced production of IL-1β in monocytes/macrophages and by other factors such as the increased production of TIMP-1 in chondrocytes (Fig. 7). Although the reduced RASF-mediated cartilage invasion is visible in the SCID mice, suggesting a protective therapeutic effect, the interaction with other cell types with an intact activin/follistatin self-regulatory cycle has to be taken into account.

## Conclusions

In conclusion, activin A reduces the invasive behavior of RASF in the SCID mouse model, indicating a possible protective role in RA. The known proliferative effects of activin A on RASF and the increase of VEGF release in vitro and the unaltered MMP and cytokine release in the presence of activin A are not able to explain the observation in vivo. However, the observed effects in the SCID mouse model could be mediated via interaction with other local cells such as macrophages. Therefore, activin A seems to be involved in the pathogenesis of RA but it plays an ambivalent role with partially pro- as well as anti-inflammatory components depending on the evaluated cell type.

## Additional files

Additional file 1: Western blot confirmed the presence of the ACVR2A receptor on RASF, which was not altered by different concentrations of activin A (10 to 30 ng/ml) after 15 h stimulation. (PPT 134 kb)

Additional file 2: Synovial fibroblast stimulation with activin A; increasing suppression of follistatin release through RASF by activin A concentrations of 0.5 ng/ml and higher (*n* = 3, experimental replicates). (PPT 220 kb)

## Data Availability

The datasets used and/or analyzed during the current study are available from the corresponding author on reasonable request.
