# Supplementary figures and images for: The activin-follistatin anti-inflammatory cycle is deregulated in synovial fibroblasts
Source: Arthritis Res Ther. 2019 Jun 10;21:144. doi: 10.1186/s13075-019-1926-7 (PMC6558802; doi:10.1186/s13075-019-1926-7)

## Slide 1
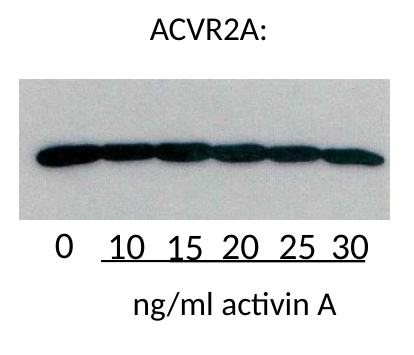

ACVR2A:
0
10
20
25
30
15
ng/ml activin A

Supplement: Supplementary file 1 — Western blot confirmed the presence of the ACVR2A receptor on RASF, which was not altered by different concentrations of activin A (10 to 30 ng/ml) after 15 h stimulation. (PPT 134 kb) [file 13075_2019_1926_MOESM1_ESM.ppt]

## Slide 1
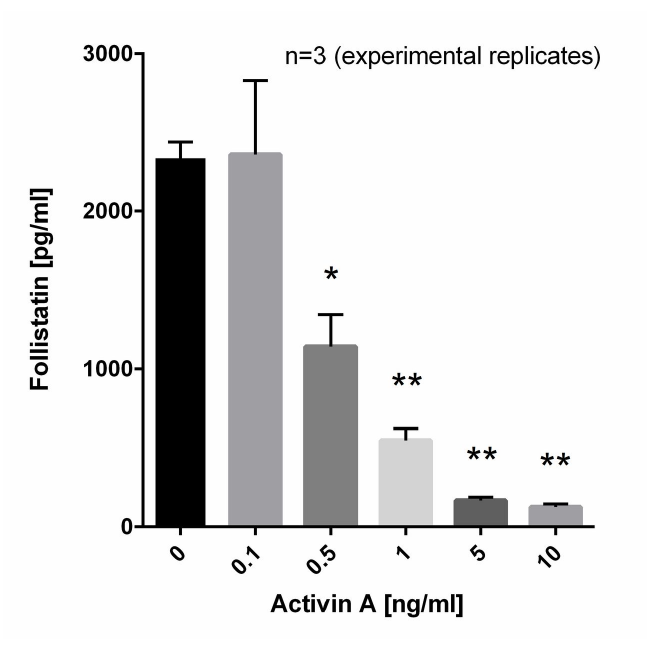

Supplement: Supplementary file 2 — Synovial fibroblast stimulation with activin A; increasing suppression of follistatin release through RASF by activin A concentrations of 0.5 ng/ml and higher (n = 3, experimental replicates). (PPT 220 kb) [file 13075_2019_1926_MOESM2_ESM.ppt]
